# Totally Extraperitoneal Herniorrhaphy (TEP): Lessons Learned from Anatomical Observations

**DOI:** 10.1155/2021/5524986

**Published:** 2021-04-21

**Authors:** Xue-Lu Zhou, Jian-Hua Luo, Hai Huang, You-Hua Wang, Huan-Bin Zhang

**Affiliations:** Department of Surgery, Chashan Hospital of Guangdong Medical University, Guangzhou, China

## Abstract

**Background:**

Totally extraperitoneal herniorrhaphy (TEP) is a therapeutic challenge because of its complex anatomical location in inguinal region. The aim of this study was to describe the related surgical anatomy through laparoscopic observation and share the lessons learned from a review of 250 primary inguinal hernia repair procedures performed at our hospital from January 2013 to November 2019. *Patients and Methods*. There were 245 men and 5 women (median age: 63.2 years). Right hernia (60.2%) was the most common site. Indirect hernia (60.5%) was the most common abnormality. The classification of type II (65.0%) was the most common form. Surgical techniques comprised retromuscular approach using cauterized dissection, management of variations of arcuate line, Retzius space and Bogros space dissection, hernia sac reduction, and mesh positioning.

**Results:**

The incidence of peritoneum injury was in 27 (10.1%). No epigastric vessels were injured. There were 8 (3%) hematoma and 18 (6.8%) seroma. No mesh infection, chronic pain, and recurrence were found after follow-up of an average of 35 months.

**Conclusion:**

A good understanding of the anatomically complex nature in the inguinal region can make it easier and safer to learn the TEP approach. Early and midterm outcomes after TEP are satisfactory.

## 1. Introduction

Totally extraperitoneal herniorrhaphy (TEP) is to use a large mesh to overlap the weakness of the myopectineal orifice (MPO) in the preperitoneal space between the peritoneum and the transversalis fascia, where direct, indirect, or femoral hernias originate. Without opening the peritoneum, TEP reduces the risk of visceral injuries, port-site hernia, and postoperative adhesion [1, 2]. Many surgeons, however, hesitate to perform TEP since the anatomy in the inguinal region is complex and the working space is narrow. Also, although rare, there is a tendency in TEP for more vascular injuries [2, 3]. Variations of the arcuate line also exist, and consequently, the preperitoneal working space may become unfamiliar [4, 5]. TEP has a longer learning curve than transabdominal preperitoneal repair (TAPP). So, knowledge of inguinal anatomy remains of greatest importance. Success in correct preperitoneal dissection depends to some extent on the existing quality of the tissue within the working area and, equally, if not more so, on the perfect technical use of these fascial structures by the surgeon.

The objective of this study is to stress the importance of a thorough understanding of the anatomy of inguinal hernia, especially as it relates to these fascial structures and the arcuate line, during TEP repair using the retromuscular approach and to summarize the lessons learned from anatomical observations.

## 2. Patients and Methods

### 2.1. Study Population

From January 2013 to July 2019, we have performed laparoscopic hernia repairs on 250 consecutive patients with primary hernias. The study included 245 males and 5 females, with an average age of 63.2 (range: 43∼78) years. The clinical data of the patients are summarized in Table 1. There was a total of 266 hernias, with 160 on the right side, 86 on the left side, and 10 bilateral sides (20 hernias). There were 161 indirect hernias, 103 direct hernias, and 2 femoral hernias. According to the classification by Nyhus et al. [6], there were 91 of type II, 173 of type III, and 2 of type IV. The study was approved by the ethics committee of the hospital, and informed consent was obtained from all individual participants included in the study.

### 2.2. Surgical Technique

We performed all operations using the posterior rectus approach (retromuscular approach) under direct laparoscopic vision with general anesthesia.

#### 2.2.1. Step One: Incision, Creating Initial Working Space, and Trocar Positioning

**Figure 1:**
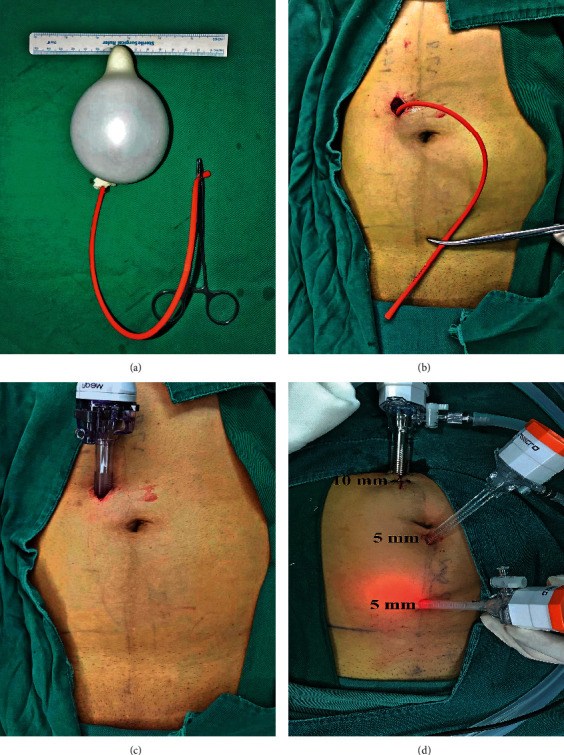
Incision, dissection of retromuscular space with handmade balloon and placement of trocars. (a) A handmade balloon which was made of a finger of glove tied to a small tube. (b) The retromuscular space was insufflated about 150 ml of air with a syringe. (c) A 10 mm trocar was inserted through incision. (d) Two other 5 mm trocars were positioned.

An incision was made 1.5 cm above the umbilicus, then extending laterally for about 2 cm. After incising the anterior rectus sheath (ARS), the rectus abdominis muscle (RAM) was retracted. The retromuscular space exposed and was separated downwards on the posterior rectus sheath (PRS) with the forefinger initially. Subsequently, we distended the superior retromuscular tunnel with a 150–200 ml home-made balloon (Figures 1(a) and 1(b)). A 10 mm trocar was inserted through the tunnel (Figure 1(c)) and pressurized to 10–13 mmHg with CO_2_. A 5 mm trocar was then inserted below the umbilicus under direct vision and another 5 mm trocar in the midway between the umbilicus and the pubic tubercle (Figure 1(d).

#### 2.2.2. Step Two: Dissecting the Preperitoneal Space

**Figure 2:**
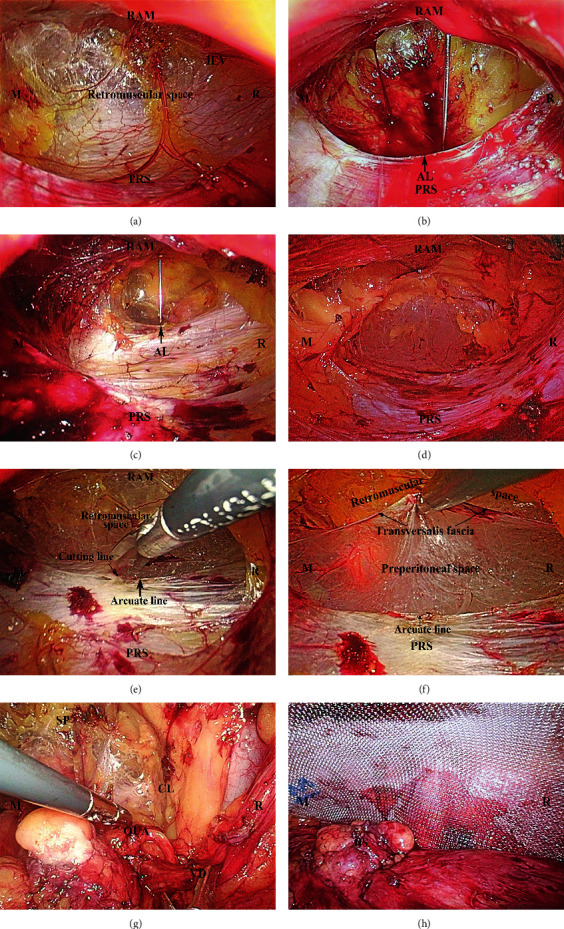
Totally extraperitoneal herniorrhaphy (TEP). (a) Dissecting the retromuscular space caudally. (b) Percutaneous needle confirmation for measuring the length of the arcuate line. Classical AL: single sharp well-defined arcuate line (4.5 cm below the umbilicus) (black arrow). (c) Low AL: multiple and low AL in the incomplete PRS (11 cm below the umbilicus) (black arrow). (d) Absent AL in the complete PRS. (e) Artificial AL was surgically created by incising PRS and the transversalis fascia beneath. (f) Retromuscular space (ventrally) and preperitoneal space (dorsally) were seen clearly. (g) The vas deferens was freed to obliterated umbilical artery. (h) Weakness of MPO was covered by a large mesh. RAM = rectus abdominis muscle, PRS = posterior rectus sheath, IEV = inferior epigastric vessels, R = right, M = middle. AL = arcuate line, SP = symphysis pubis, CL = Cooper's ligament, VD = vas deferens, OUA = obliterated umbilical artery, and HS = hernia sac.

In order to decrease bleeding caused by tiny blood vessels between RAM and PRS, the space was caudally dissected along the surface of PRS with electrocautery (“sweeping the floor maneuver”) until the junction of the arcuate line and the transversalis fascia (Figure 2(a)). At this stage, we measured the length of the arcuate line by percutaneous needle positioning at relevant landmarks (Figure 2(b)). We have interestingly observed three common types of arcuate line: the first was referred to as the classical arcuate line (4-5 cm below the umbilicus), whose PRS was acutely demarcated over the transversalis fascia, creating a clear border with the arcuate line (Figure 2(b)); the second was called low arcuate line (6–11 cm below the umbilicus), for the attenuated fibers of PRS gradually disappeared over the transversalis fascia (Figure 2(c)); and the last was absent arcuate line, where the thickened complete PRS formed by both the posterior rectus sheath and the transversalis fascia extended up to the pubic bone (Figure 2(d)). For those of the classical type, the arcuate line was incised in order to enter the space of Retzius and Bogros, and then the dissection was carried out caudally. Here, inferior to the arcuate line, we observed that the rectus abdominis rests directly on the transversalis fascia. While for those patients with variation of arcuate line (or very low type or absent type), an artificial arcuate line necessitated to be created through incising both PRS and the transversalis fascia transversely at a convenient place to enter the preperitoneal space correctly (which is described in another article) (Figure 2(e)). In this way, the proper preperitoneal space characterized by the “spiderweb-like” loose areolar tissue can be entered properly, which leads to the avascular space of Retzius (Figure 2(f)). It was obvious that, unless this was done, the space can be only retromuscular space (instead of preperitoneal space), and the risks of complications and recurrence may be increased.

#### 2.2.3. Step Three: Dissecting the Medial Compartment (Retzius Space)

Continuing from the preperitoneal space, the Retzius space was dissected among the transversalis fascia, the superficial layer of preperitoneal fascia (or membranous layer of the extraperitoneal fascia), and medial to epigastric vessels. The pubic symphysis, Cooper's ligament, and corona mortis were identified. A femoral or direct inguinal hernia is easily identified and reduced in this medial compartment during this stage. A direct hernia sac greater than 5 cm was inverted by suturing to the pubic ramus [2, 7].

#### 2.2.4. Step Four: Dissecting the Lateral Compartment (Bogros Space)

Bogros space lateral to the Retzius space then was freed between the peritoneum and the deeper layer of the preperitoneal fascia (or inner fatty layer of extraperitoneal fascia) by sweeping movement, laterally towards the anterior superior iliac spine. The lateral arcuate line near the linea semilunaris was also often incised for optimal mesh placement, especially in the presence of a low arcuate line, to expand the working surgical field [8].

#### 2.2.5. Step Five: Reduction of Indirect Hernia Sac and Parietalization of Spermatic Cord

Between the spaces of Retzius and Bogros, there lies only the conical structure of the spermatic cord and the indirect hernia enclosed by it. The internal spermatic fascia was entered by incision or blunt separation of its fibers. The indirect hernia sac was then reversed by gently pulling the peritoneum cranially [9, 10]. As for very large or scrotal hernias, the hernia sac can be divided at the level of the internal ring, and the redundant sac can be left in the inguinal canal after hemostasis. The continuity between these two spaces (or different anatomical planes of the preperitoneal fasciae) was established by resecting the interfoveolar ligament, creating a preperitoneal space big enough to accommodate the mesh. The vas deferens should be dissected until the obliterated umbilical artery is encountered (Figure 2(g)). And, the testicular vessels should be stripped cranially at least 6 cm from the internal ring. Before accommodation of the mesh, a final systematic inspection should be carried out. Any tissue that prevented the observation of Cooper's ligament was a medial or femoral hernia and should be reduced. Any larger peritoneal tear should be repaired promptly to prevent adhesion of abdominal viscera to the mesh.

#### 2.2.6. Step Six: Positioning the Mesh

The hernia repair was achieved using a large mesh (3 D of 10.8 cm × 16 cm; Bard Davol Inc. Warwick, RI, 02886, USA) to completely cover MPO with overlap between transversalis fascia and the preperitoneal fascia like a sandwich (Figure 2(h)). As Bard 3 D Max conforms and compresses to the shape of the mesh and the pelvic wall, the mesh was not fixed, instead relying on the pressure of abdominal cavity to keep it in position.

#### 2.2.7. Step Seven: Surgical Egress

After withdrawing trocars, the anterior rectus sheath was stitched with absorbable suture. The skin incisions were closed with suture.

## 3. Results

Demographics and hernia characteristics of the patients are shown in Table 1. A total of 249 patients successfully underwent TEP repair. During the procedure, we had 26 minor peritoneal injuries which needed to be adequately sutured and one large tear which led to immediate conversion. No epigastric vessels were damaged. The classical type of arcuate line accounted for 16.8% (42/250) of the variations, the low type, 36.0% (90/250), and the absent type, 47.2% (118/250).  Table 2 shows postoperative complications. Of all, 18 patients had acute urinary retention after general anesthesia, which needed catheterization for a few days. There were eight cases of hematoma: seven of them required aspiration and one needed scrotal drainage. Sixteen cases of seroma were found: six needed an aspiration and ten others resolved spontaneously. One patient had a foreign body sensation in the right groin, and the symptom disappeared after six months. There was no infection, chronic pain, or recurrence after a mean follow-up of 35 months.

## 4. Discussion

In our experience, TEP, using a large mesh, has been demonstrated to be excellent for the correction of an inguinal hernia in adults after midterm follow-up. The outcomes provided a low risk of postoperative complications (9%) related to the TEP approach. There was no chronic pain and recurrence.

The success of TEP depends on many factors, including the characteristics of the hernia, the body habitus of the patients, and mainly surgeon's knowledge of the anatomy in inguinal correct dissection. The lessons learned from our experience offered some advantages.

**Figure 3:**
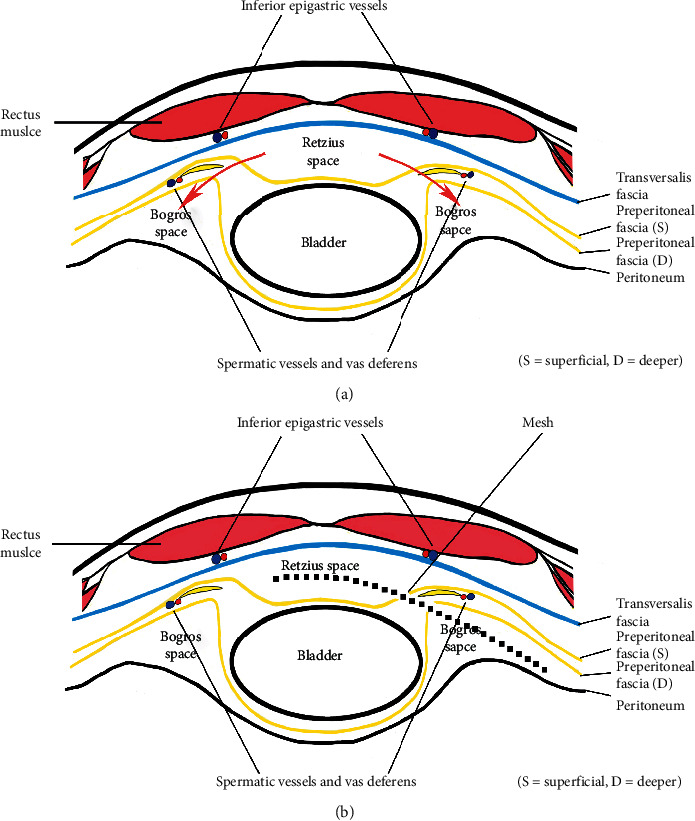
Schematic illustration of a horizontal section of the lower abdominal wall (Retzius' and Bogros' space). (a) The red arrows illustrated the approach path into the Bogros space, which communicated these two discontinuity planes for spreading the position of mesh. (b) The position of mesh (dashed line), i.e., placing between the transversalis fascia and the superficial layer of preperitoneal fascia in the Retzius' space, the deeper layer of preperitoneal fascia, and the peritoneum in the Bogros' space.

First, the incision mentioned above made a valuable perspective effect on the PRS and facilitated the repair of bilateral hernias. Second, we utilized handy balloon to insufflate only the upper aspect of the retromuscular plane, creating initial working space, and then we can caudally dissect the space along the PRS by electrocautery. In this way, under direct vision, we can maintain the integrity and clarity of the PRS, and we were able to not only decrease oozing from tiny vessels caused by blind telescope dissection but also reduce risk of damaging inferior epigastric vessels underneath the rectus. Ren et al. [11–14] reported that the incidence of vessel injury during TEP was 1.3∼36.3%, which was much higher than that during TAPP (0.8%). They pointed out that the main reason for the injury was that the blind dissection did not enter correct preperitoneal space. If inferior epigastric artery was injured inadvertently, blood loss could reach hundreds of milliliters or even thousands of milliliters which threatened life [15]. In our series, however, there was no injury of inferior epigastric vessels and their branches by using this unique technique. On the other hand, through this well-established tunnel, the morphology of the arcuate line and its variations was able to observe clearly, which were confirmed and described as above. In this stage, the following anatomical landmarks can be identified: RAM, PRS, arcuate line, epigastric vessels, and so on. Third, as we all know, the basic principle of TEP is to accommodate a large mesh in the preperitoneal space. Before entering it, however, it is necessary first to refer briefly to the anatomy of the arcuate line and the transversalis fascia. According to the textbook definition, the arcuate line of the abdomen, also referred to as linea semicircularis or Douglas' line, is a horizontal line that demarcates the lower limit of the posterior layer of the rectus sheath. It occurs 4-5 cm below the umbilicus. The transversalis fascia lies deep to PRS, below the arcuate line, and the rectus abdominis rests directly on the transversalis fascia. However, this classic arrangement has been challenged in the anatomic and clinical literature [5, 16]. The transition zone between the arcuate line and the transversalis fascia, as we conceived it, was the open “gate” for gaining access to the preperitoneal space. For those of classical type, the preperitoneal space was entered simply by incising the arcuate line, while for those of low type and absent type, one needed deliberately to create an artificial arcuate line on the posterior rectus sheath in order to enter the preperitoneal space. After this, it can be observed that the transversalis fascia delineates two important surgical planes between the rectus and the peritoneum (Figure 2(f)). The retromuscular plane, between the rectus and the transversalis fascia, contains the inferior epigastric vessels, while the preperitoneal plane, between the transversalis fascia and the superficial layer of the preperitoneal fascia, contains the median and medial umbilical ligaments enveloped by the visceral fascia of the bladder. This is the ideal plane of dissection, leading caudally to the Retzius' space. Embryologically, the preperitoneal fascia originates from the mesoderm, expanding between the ectoderm (transversalis fascia) and endoderm (peritoneum). The bladder, vas deferens, and testicular vessels are enveloped by the superficial and deeper layers of the preperitoneal fascia, which extends to envelope the ureter, kidney, and adrenal gland by posterior and anterior leaves of renal fascia. Accordingly, Retzius' space lies between the transversalis fascia and the superficial layer of preperitoneal fascia [17–21] (or membranous layer of the extraperitoneal fascia [22, 23] (Figure 3(a)). Consequently, the dissection of Retzius' space should be carried out between these two fasciae.

Even now, however, controversy remains about the exact fascial anatomy and Retzius' space embedded by them. Therefore, more information, including a cadaver study, is needed to clarify the relationship of fasciae and Retzius space in between. Also, in this anatomical plane, landmarks, including the pubic bone, Cooper's ligament, epigastric vessels, corona mortis, and iliopubic tract, can be palpated or inspected.

Unlike the anatomical plane of Retzius mentioned above, Bogros' plane was located between the peritoneum and the deeper layer of the preperitoneal fascia (or inner fatty layer of extraperitoneal fascia) [22, 23] (Figure 3(a)). This division of the preperitoneal space may be attributed to the bladder being enveloped by the superficial and deeper layer of the preperitoneal in the medial compartment, but there being no equivalent organ present in the lateral compartment. Therefore, the peritoneum in the Bogros' space is in direct contact with the deeper layer of the preperitoneal fascia, from which the Bogros' space can be peeled open by freeing the peritoneum (using a sweeping motion).

The spaces of Retzius and Bogros are separated by a thickened band of peritoneum or subperitoneal fibrous tissue, depending on the patient's body habitus and fat distribution. It is seen going from an anterior and lateral attachment to the abdominal wall and travelling to a posterior and medial connection to the bladder in TEP anterior view. Obviously, if this connective tissue (or interfoveolar ligament) along the path of the epigastric vessels was deliberately incised, the two spaces can be communicated to create a big enough preperitoneal space (Figure 3(b)). The mesh should be placed in both the medial compartment—between the transversalis fascia and the superficial layer of the preperitoneal fascia—and the lateral compartment—between the peritoneum and the deeper layer of the preperitoneal fascia (Figure 3(b)). The nerves in the “triangle of pain” are well protected by the preperitoneal fatty tissue which remains undisturbed, so this can decrease the risk of postoperative chronic pain resulting from irritation of the overlying mesh. Once dissection of Retzius' and Bogros' spaces is complete, the triangular structure of the cord (bounded laterally by spermatic vessels, medially by vas deferens, and superiorly by internal ring) becomes visible clearly. The hernia sac can only be reduced or transected if the internal spermatic fascia is incised [8, 9, 17]. The divided stump of a larger sac should be thoroughly coagulated to prevent it from bleeding. In our series, the incidence of hematoma was a little higher (3%), and the reason may be related to the proportion of larger scrotal hernias. Then, the parietalization of the spermatic cord was sufficiently extended at least 6 to 8 cm from the internal ring. And, lastly, a “no-touch technique” was needed in order to reduce the risk of mesh infection. The mesh was positioned as described, but was not fixed because Bard 3 D Max conformed to the shape of the mesh and the pelvic wall. The main advantage of nonfixation is the reduced incidence of postoperative pain [2, 24].

In conclusion, a good understanding of the anatomically complex nature of the inguinal region can make it easier and safer to learn the TEP approach. We have found this technique extremely useful in the course of over 250 laparoscopic hernia repairs.

## Figures and Tables

**Table 1:** Demographics and hernia characteristics of the patients.

No. of patients (no. of hernias)	250 (266)
Mean age (range)	63.2 (43–78)
Mean body mass index	24.2 ± 2.33

*Sex*	
Male	245 (98%)
Female	5 (2%)

*Location of hernia*	
Right	160 (60.2%)
Left	86 (32.3%)
Bilateral	20 (7.5%)
Mean operative time (range)	63 (55–90 min)
Mean hospital stays (range)	7 (5–10 d)
Mean follow-up (range)	35 (6–70 mo)

**Table 2:** Intraoperative and postoperative complications.

Intraoperative	
Peritoneal tear	27 (10.1%)
Inferior epigastric vessels injury	0 (0%)
Bladder injury	0 (0%)
Postoperative	
Hematoma	8 (3.0%)
Seroma	16 (6.0%)
Wound infection	0 (0%)
Chronic pain	0 (0%)
Urinary retention	18 (6.8%)
Recurrence	0 (0%)

## Data Availability

The data generated during this study are available from the corresponding author upon request. The patients' data, however, cannot be made available.
